# The service user experience of SlowMo therapy: A co‐produced thematic analysis of service users’ subjective experience

**DOI:** 10.1111/papt.12393

**Published:** 2022-04-20

**Authors:** Kathryn E. Greenwood, Megha Gurnani, Tom Ward, Evelin Vogel, Claire Vella, Alison McGourty, Sam Robertson, Catarina Sacadura, Amy Hardy, Mar Rus‐Calafell, Nicola Collett, Richard Emsley, Daniel Freeman, David Fowler, Elizabeth Kuipers, Paul Bebbington, Graham Dunn, Daniel Michelson, Philippa Garety, the SlowMo Patient, Public Involvement (PPI) team

**Affiliations:** ^1^ School of Psychology University of Sussex Brighton UK; ^2^ Sussex Partnership NHS Foundation Trust Worthing UK; ^3^ Department of Psychology Institute of Psychiatry, Psychology and Neuroscience King’s College London London UK; ^4^ South London and Maudsley NHS Foundation Trust London UK; ^5^ Oxford Health NHS Foundation Trust Oxford UK; ^6^ Department of Biostatistics and Health Informatics Institute of Psychiatry, Psychology and Neuroscience King’s College London London UK; ^7^ Department of Psychiatry Oxford University Oxford UK; ^8^ Division of Psychiatry University College London London UK; ^9^ Centre for Biostatistics School of Health Sciences Manchester Academic Health Science Centre The University of Manchester Manchester UK

**Keywords:** cognitive behaviour therapy, paranoia, patient and, psychosis, public involvement, qualitative, service user experience, schizophrenia‐spectrum, reasoning, thematic analysis

## Abstract

**Objectives:**

SlowMo is the first blended digital therapy for paranoia, showing significant small‐moderate reductions in paranoia in a recent large‐scale randomized controlled trial (RCT). This study explored the subjective service‐user experience of the SlowMo therapy content and design; the experience of the blended therapy approach, including the triangle of the therapeutic alliance; and the experience of the digital aspects of the intervention.

**Design:**

Qualitative co‐produced sub‐study of an RCT.

**Methods:**

Participants were 22 adult service users with schizophrenia‐spectrum psychosis and persistent distressing paranoia, who completed at least one SlowMo therapy session and a 24‐week follow‐up, at one of 3 sites in Oxford, London, and Sussex, UK. They were interviewed by peer researchers, using a topic guide co‐produced by the Patient and Public Involvement (PPI) team. The transcribed data were analysed thematically. Multiple coding and triangulation, and lay peer researcher validation were used to reach a consensus on the final theme structure.

**Results:**

Six core themes were identified: (i) starting the SlowMo journey; (ii) the central role of the supportive therapist; (iii) slowing things down; (iv) value and learning from social connections; (v) approaches and challenges of technology; and (vi) improvements in paranoia and well‐being.

**Conclusions:**

For these service users, slowing down for a moment was helpful, and integrated into thinking over time. Learning from social connections reflected reduced isolation, and enhanced learning through videos, vignettes, and peers. The central role of the supportive therapist and the triangle of alliance between service user, therapist, and digital platform were effective in promoting positive therapeutic outcomes.

**Practitioner points**

- Service users with psychosis are able to use technology in therapeutic settings, with clinician support, to reduce paranoia and enhance well‐being outcomes in daily life. This does suggest that therapies for people with psychosis can be improved by blending digital technology with face‐to‐face sessions.
- The role of the supportive therapist is central to the triangle of alliance between therapist, service user, and digital platform in blended therapy delivery for psychosis: The therapist aids engagement with the therapy and the technology; whilst the digital components reinforce the therapy.
- Slowing down for a moment is a core active element of the SlowMo therapy to reduce paranoia, but learning to slow down ones thinking process takes time and practice.
- The opportunity to learn from the vignettes and video stories of peers with the same lived experience was also critical to the experience. For some, this importantly reduced the sense of isolation as people realized they were not alone with their experiences.

## INTRODUCTION

Paranoia, or fear of harm from others, is common, occurring frequently in as many as 20% of the general population and up to 70% of people with non‐affective psychosis, and severely impacting mental health and quality of life (Coid et al., 2013; Freeman & Garety, 2006; Freeman et al., 2011; Johns et al., 2004). Yet there are issues with the delivery and uptake of standard psychological therapies for paranoia and psychosis (Greenwood et al., 2018). In addition, meta‐analyses of traditional cognitive behaviour therapy (CBT) for psychosis therapies have revealed only small–moderate effects on delusions and positive symptoms (e.g. Bighelli et al., 2018). Small to moderate treatment effect sizes have also recently been found for the impact on the paranoia of briefer causal‐interventionist therapies that specifically target sleep, worry, and self‐schema, with much greater effects on the specifically targeted process (Freeman et al., 2014, 2015a, 2015b). However, the psychological mechanisms underpinning paranoia, also comprise reasoning biases of jumping to conclusions (fast decision making based on limited evidence), and reduced belief flexibility (meta‐cognitive ability to reflect on and adjust beliefs) (Garety & Freeman, 2013; Garety et al., 2005; Ward & Garety, 2017). Targeting these mechanisms, in a brief engaging, digitally supported intervention might enhance both engagement and paranoia outcomes.

SlowMo therapy takes a causal‐interventionist approach, focusing on underlying causal mechanisms and is the first blended (therapist delivered and technology‐enhanced) digital therapy for paranoia. It involves 8 cognitive‐behavioural sessions that target ‘fast thinking’ reasoning biases that are thought to underlie paranoia (Garety et al., 2015), by encouraging people to slow down for a moment and find ways of feeling safer. Sessions are assisted by the SlowMo ‘webapp’, delivered via touchscreen laptop, which has interactive features including animated vignettes and personalized thought ‘bubbles’, and by a mobile phone app which provides access to SlowMo strategies in daily life. The therapy is brief, so has the potential to support access to therapy for a larger number of service users than current provisions, and is designed in line with recommendations for improving implementation of digital therapies for psychosis (Allan et al., 2019; Berry et al., 2019; Sarkar et al., 2016). The structured blended approach, with face to face sessions supported by session‐by‐session digital content reduces the risk of therapist drift (Kooistra et al., 2016), and the inclusive human‐centred‐design (Hardy et al., 2018), aims to ensure the therapy is usable, trustworthy, enjoyable, personalized, normalizing, and offers flexible interpersonal support. The recent large‐scale SlowMo randomized controlled trial clearly indicated that SlowMo was beneficial, with 10 /11 paranoia measures at 12 weeks, and 8/11 at 24 weeks demonstrating significant small‐moderate effects in 181 participants with schizophrenia‐spectrum psychosis in London, Sussex, and Oxford compared to 180 participants who received usual care alone (Garety et al., 2017, 2021). Improvements were also found on measures of self‐esteem, worry, well‐being, and quality of life.

In addition to the positive effects on paranoia and well‐being, understanding service user experiences of therapy are also critical. The therapeutic alliance is one aspect of the service user experience, which can have a causal impact on the effectiveness of treatments in psychosis (Goldsmith et al., 2015). Indeed, alliance with online digital interventions may promote better engagement (Clarke et al., 2016). Historically, the therapeutic alliance is defined as the quality of the working relationship, or connection, between service user and therapist, that aims to produce positive therapeutic outcomes, through shared goals, tasks, and bonds characterized by interest, warmth, empathy, authenticity, genuine concern, understanding, and hope (Bordin, 1979; Henson et al., 2019; Lopez et al., 2019). In blended therapies, this working relationship is extended to a ‘triangle of alliance’ between the service user, clinician, and digital platform (Cavanagh, 2010); the digital therapeutic alliance being the therapeutic connection between the user and the mental health app (D'Alfonso et al., 2020). The working relationship with the therapist remains a predictor of change (Vernmark et al., 2019) but the alliance can be further enhanced by the incorporation of technology, leading to greater commitment to goals and tasks (Lopez, 2015). Alliance varies across platforms and levels of therapist involvement, and alliance with digital platforms independently predicts engagement and outcome (Cavanagh et al., 2018). In SlowMo therapy, the triangle of the alliance is extended to include both the service user and therapist connection with the web app, but also the mobile phone app within and outside of sessions.

Data on the user experience of digital therapy in psychosis is only recently emerging, focused largely online and the mobile phone supported self‐management (Berry et al.,^2016^). Service users with psychosis have reported a sense of autonomy and control while using self‐guided digital health apps to manage their symptoms (Berry et al., 2019). A proof‐of‐concept trial found high feasibility and acceptability, and large treatment effects for a self‐guided smartphone app (Acticisst) compared to a symptom tracking app (Bucci et al., 2018a), whilst a smartphone and web‐based cognitive self‐management app (FOCUS) found that 87% of app users found it easy to use and 87% found it helpful for symptoms, but some reported difficulties with the technology and requested more technical support (Ben‐Zeev et al., 2014).

There is still much to be understood about the implementation of digital interventions for psychosis. Whilst implementation frameworks have been created (Allan et al., 2019), no study to our knowledge has yet evaluated a blended therapy with digitally supported face‐to‐face therapy sessions for paranoia. Research has demonstrated excellent adherence to SlowMo sessions and the mobile phone app, and self‐reported ease‐of‐use, usefulness, and enjoyment (Garety et al., 2021; Hardy et al., 2022). SlowMo therapy design was able to bridge the ‘digital divide’ (Robotham et al, 2016), whereby people from minority populations report lower digital literacy, as age and ethnicity did not impact user experience (Hardy et al., 2022). Given recommendations from researchers to employ multidimensional objective and subjective assessments of user experience to support implementation, in light of suboptimal uptake and use of mental health apps (Ng et al., 2019), this qualitative study evaluates service users’ subjective experience of and alliance with the SlowMo therapy, including therapist, digital elements, and their interaction.

## AIM

The study aimed to explore the subjective service user experience of:

- The SlowMo therapy content and concepts;
- The blended therapy approach (the triangle of alliance); and
- The digital aspects of the intervention to inform improvements prior to future implementation.

## METHOD

### Study design and setting

The study is a co‐produced thematic analysis of service user experiences of SlowMo therapy in UK mental health services. The data collection, analysis, and interpretation were co‐produced with peer researchers, to enhance the methodological rigour and provide a rich subjective perspective, as service users may be more forthcoming when interviewed by peers (Simpson & House, 2002), and peer researcher involvement in the analysis may lead to the identification of novel themes and reflections (Gillard et al., 2010, 2012). Further details of the Patient and Public Involvement (PPI) processes are described in Greenwood et al., (2021).

### Participants

Participants were 22 service users recruited for the SlowMo RCT at the 3 sites (London, Sussex, and Oxford). Sampling was consecutive and purposive: participants were invited to take part in the qualitative sub‐study consecutively, following completion of (i) at least one SlowMo session and (ii) the 24‐week follow‐up. Additional inclusion criteria were the same as for the main trial: aged ≥18 years; persistent (3+ months) distressing paranoia (assessed using the Schedules for Clinical Assessment in Neuropsychiatry [1992]; scoring >29 on the Green et al. (2008), Paranoid Thoughts Scales (GPTS – part B) [2008]; schizophrenia‐spectrum psychosis (F20‐29, ICD‐10) [2010]; capacity to provide informed consent; sufficient spoken English to participate. Exclusion: profound visual or hearing impairment; inability to engage in assessments; currently receiving psychological therapy for paranoia; primary diagnosis of substance abuse or personality disorder, organic syndrome, or learning disability.

### Topic guide

A topic guide for the qualitative interviews was initially drafted by the study team to enable a detailed evaluation of the user experience of the therapy, therapist, and technology. It was revised iteratively in collaboration with the SlowMo PPI team, initially in 2 central PPI meetings to which all 9 PPI members were invited (6 women and 3 men, from Sussex (3), Oxford (2), and London (4)), and subsequently in a series of local PPI meetings, by the PPI interviewers (1 woman in Oxford, 1 woman/1 man in Sussex, and 1 woman in London), who revised it further for flow and ease of use. See Appendix 1 for the final topic guide.

### Procedure

PPI team members received either the SlowMo therapy as part of a previous project, or an introduction to the therapy and materials at the start of the current project, to enable them to better understand and contextualize the interview discussions. They also received training in peer researcher roles and role‐play practice in conducting qualitative interviews using the topic guide. Supervision was provided by the PPI lead (Sussex) or trial coordinators (London/Oxford). After gaining additional written informed consent for participation in the qualitative study, all interview data were collected in‐person, either by 2 peer researchers working together (Sussex) or by 1 peer worker and a graduate researcher (London and Oxford). Participants were reimbursed £20 for their time. All interviews were audio‐recorded with the exception of one (see below) and transcribed verbatim.

### Analysis

A thematic analysis was applied to the transcribed data, using a constructionist framework in 6 steps: familiarization, initial coding, searching for themes, reviewing themes, defining, and naming themes, and producing the report (Braun & Clarke, 2006). The data were analysed in two phases: each comprised 11 transcripts, and used multiple coders and triangulation to reach a consensus on theme structure. In the first phase, an independent graduate psychologist, clinical, and peer researchers, and site co‐ordinator/trial therapists each coded the same transcript and met to agree with the initial coding frame. Further transcripts were coded by the graduate psychologist and clinical researcher. Codes were summarized and reviewed by the wider group to clarify the theme structure. The graduate psychologist and clinical researcher then coded the remaining transcripts to produce the phase 1 theme structure. The second phase was conducted by a second graduate researcher, mirroring the phase 1 approach. The two coding frames were then combined. Discrepancies between phases 1 and 2 were discussed, quotes were reviewed, and the consensus was reached on theme descriptors. Finally, themes and quotes were presented to the whole PPI team in two meetings across all sites for final validation.

### Epistemological position

Lack of consistency and coherence in thematic analysis (Holloway & Todres, 2003) can be reduced through the transparent application of an epistemological position that underpins study findings (Nowell et al., 2017). The study is underpinned by a critical realist perspective (Bhaskar, 2009; Collier, 1994), which recognizes that whilst reality exists, knowledge is socially produced and influenced by the observer's context and worldview (Bhaskar, 2009; Ponterotto, 2005). There may be multiple equally valid accounts of the same phenomenon (Hammersley, 2004) such that reality is only imperfectly known (Letourneau & Allen, 2006; Porter, 2007). Multiple approaches were taken to best capture the reality of participant perspectives, including data collection by peer researchers, multiple coding of themes from different perspectives, triangulation which is a process of discussion to reach consensus on theme structure involving all peers and researchers, final validation, and transparency in reporting.

### Stance of the PPI peer researchers

The peer researchers were not experienced in qualitative research and brought a ‘lay’ perspective to the approach, but they had worked as SlowMo PPI members for 1.5 years before they commenced data collection, and 2 of the 4 peer researchers had received a version of SlowMo therapy. This enabled them to empathize with participants’ experiences but may have contributed bias in their interpretation of results.

### Stance of the graduate coders

The primary coder in phase 1 was independent of the SlowMo study, which enabled objectivity and reduced the risk of bias, but also limited understanding of participants’ responses, and required discussion with the broader study team. The primary coder in phase 2 was a SlowMo graduate research assistant, who was familiar with the intervention and the phase 1 coding but undertook phase 2 coding separately with a critically reflective stance.

## RESULTS

Of 28 eligible participants approached in Sussex and London, 5 declined to participate, and 3 were unreachable. One did not consent to audio‐recording, and notes were taken during their interview instead. Two additional participants took part from Oxford to give 22 participants, comprising 12.2% of the total SlowMo therapy sample. Basic demographic details are provided in Table 1. Some data are not presented individually to preserve anonymity. Eighteen were male (82%), 18 were White British (82%), 19 were single (86%), 17 were unemployed (77%), and 14 lived alone (64%). Only 3 participants (14%) had received education beyond A levels. Their mean age was 44.9 years (range 20–64), and their mean GPTS Part B score was 51.8 (range 29–79). The qualitative sample was broadly representative of the whole therapy sample, which had slightly fewer men (70%), slightly more ethnic diversity (70%) and unemployment (80%), slightly fewer people living alone (58%), a slightly lower average age (42.6), and slightly higher GPTS Part B score (56.2) (Garety et al., 2021).

**TABLE 1:** Participant demographics

	Age	Gender	Highest education	Working status	Living situation
*Sussex*					
*S1*	20	M	Secondary (O/CSE)	Unemployed	Parents
*S2*	61	M	Secondary no exams	Unemployed	Others
*S3*	57	M	Secondary no exams	Unemployed	Alone
*S4*	46	F	Higher education	Unemployed	Alone
*S5*	42	M	Higher education	Volunteer	Parents
*S6*	30	M	Secondary no exams	Unemployed	Alone
*S7*	27	M	Vocational/college	Unemployed	Alone
*S8*	53	M	Secondary (O/CSE)	Unemployed	Alone
*S9*	39	M	Secondary (A level)	Part‐time	Alone
*S10*	58	M	Secondary (O/CSE)	Unemployed	Alone
*S11*	64	M	Secondary no exams	Unemployed	Alone
*S12*	49	M	Secondary (O/CSE)	Volunteer	Alone
*Oxford*					
*O13*	34	F	Higher education	Part‐time	Others
*O14*	47	M	Secondary (O/CSE)	Unemployed	Alone
*London*					
*L15*	24	M	Secondary (O/CSE)	Unemployed	Parents
*L16*	43	M	Secondary (O/CSE)	Unemployed	Others
*L17*	56	F	Primary school	Unemployed	Alone
*L18*	35	M	Secondary (O/CSE)	Volunteer	Parents
*L19*	54	M	Vocational/College	Unemployed	Alone
*L20*	31	M	Vocational/College	Unemployed	Relatives
*L21*	63	F	Secondary (O/CSE)	Unemployed	Alone
*L22*	54	M	Secondary (O/CSE)	Unemployed	Alone

Note

Ethnicity and marital status data have been removed to preserve anonymity for people who may have been in a minority status on a particular site.

### Development and validation of theme structure

The final theme structure comprised 6 core themes and 20 sub‐themes. Two core themes remained unchanged from phase 1 to 2 (‘Slowing things down’ and ‘Improvements in paranoia and well‐being’), the wording changed slightly for one (‘Starting the SlowMo journey’); the wording and focus (changed slightly for another ‘Approaches and challenges of Technology’). The final two themes became more distinct: ‘Feeling connected and understood’ became ‘Value and learning from social connections’; ‘Drivers of Progress’ became specifically, ‘The central role of the supportive therapist relationship’. Of the final 20 sub‐themes, 6 were identical from phase 1 to phase 2, 9 changed wording or focus, and 5 were new in phase 2.

**FIGURE 1:**
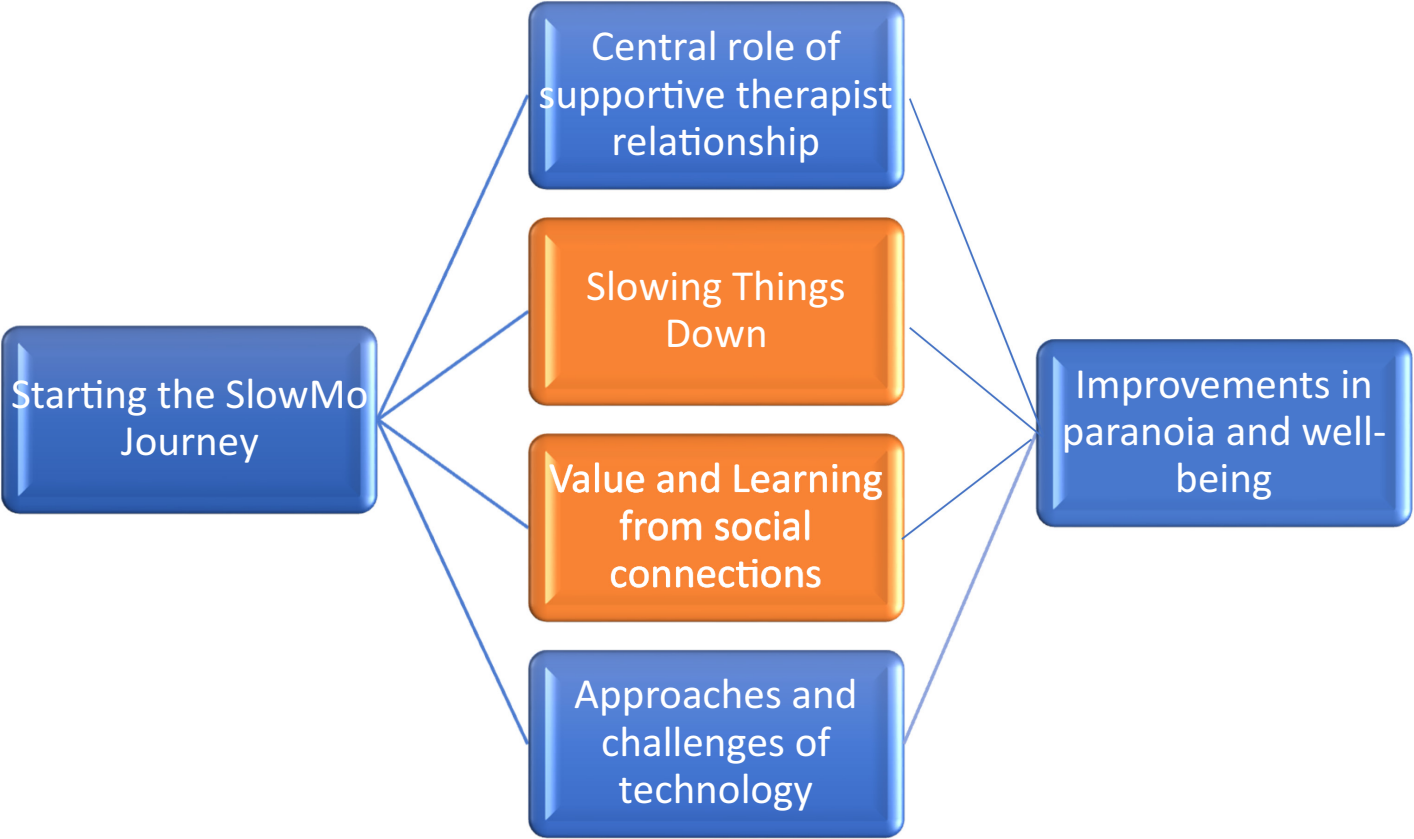
The structure of qualitative experiences of SlowMo

Figure 1 provides a representation of the organization of the core themes. The initial theme captures the experience of (i) starting the SlowMo Journey. The inner two themes, depicted in orange, reflect core and unique elements of the SlowMo therapy as perceived by service users: (iii) slowing themes down and (iv) value and learning from social connections. The two flanking themes, (ii) the Central role of the supportive therapist relationship and, (v) approaches and challenges of technology, emphasize the critical connections with both therapist and technology in leading to the therapeutic outcomes of (vi) Improvements in Paranoia and well‐being.

Supporting quotes for all themes and sub‐themes are provided in Table 2.

**TABLE 2:** Participant quotes to support themes

1. Starting the SlowMo journey	1.1 Reasons for starting SlowMo	S8: ‘Oh mainly, cause I wasn't getting anywhere, my illness or whatever, has been on the same tablets for a long time. I wasn't getting anywhere so I thought I might try something’ L17: ‘It was [CPN] that got me to do it, she came to my house then she showed me this leaflet. She said, You've got a 50/50 chance of doing it, would you like to?, I said, “I’ll try, I’ll have a go,”’
	1.2 Feelings before starting	L21: ‘I was nervous, very nervous. I didn't have no confidence, so I have now…’ O14: ‘Apprehensive. Meeting with people I didn't know’ O13: ‘I am eager to explore new opportunities as well, so that kind of made me go for it.’
	1.3 Voices and anxiety as barriers	S10: ‘It was daunting at first, cause it was the first time I’d actually spoken about them yeah. The way I felt with my voices. Basically it took me a good 10 minutes to listen to what she was saying because my voices were telling me not to listen’
2. Central role of supportive therapist relationship	2.1 Importance of talking and being listened to	L16: ‘Just being able to talk about my problems and focus on them and just come up with genuine ideas’ O13:’I found the talks we had …most invaluable. I thought that was amazing …actually having someone to talk to when we were going through it’
	2.2 A positive therapist relationship	L21: ‘He made me really relaxed. Any problems I had, I could talk to him, which I don't usually … So things came out that I haven't told anybody’
	2.3 Therapist as a supportive guide	S4: ‘I trusted her and felt comfortable with her. She explained everything very clearly… she was very sensitive towards my feelings when I was struggling …and with the computer she just helped me to see things more clearly’ O13: ‘I think if it was just the computer, then again it wouldn't work, I think you need that interaction as well… I had one bad day where I just couldn't focus and that was just really bad. But then I had someone who was really understanding about that, and that made all the difference, and I didn't feel like too pressured. I could learn at my own pace’ L21: ‘Never used a computer, but I learnt with [therapist]’ S5: ‘The computer was really there to support the face‐to‐face talk. Uhm. It seemed to me that the therapy had been done so that it gave equal problems to the face to face therapy and computer therapy…. I preferred in some ways more emphasis on the face to face … if it's more psychological therapy which involves emotions… having a computer may sort of negate that, make it feel bland. Uhm. But in the way it's done with [therapist], it seemed to work quite well’
3. Slowing things down		L18: ‘It was very introductory kind of thing. I felt maybe quite appropriately it was quite slow actually to get to the juicy part if you like. And [therapist] would say I was often ahead of the project, ahead of the sessions because I was thinking of things that were going to be introduced later on. So …for someone who is sort of getting to grips with it a bit quicker it could be condensed or you could introduce sort of the more advanced part earlier on’
	3.1 Slow and fast thinking are relevant and helpful	L17: ‘I don't worry so much, it's my neighbours, they make me stressed and then I’ll say,“No, I’ve got to slow down.” You have to, because if not, if you carry on, you make yourself ill and you'll land up in hospital’ O14: ‘I always insisted on going on … a split‐second decision …, which is basically fast thinking. I was trained to always look out for the worst case scenario … SlowMo slow thinking wasn't difficult, but it was different … I found that using that where I live, all those idiots in the other blocks, if you think through possible other scenarios and then think, “I don't actually know those people and they don't know me, so they can't be talking about me,” whereas prior to SlowMo I would think, “Why are they talking about me? What's going on?” and that would stress me out really badly’
	3.2 Learning a new thinking skill set	L21: ‘It was very hard because I think quickly, but I slowed it down and I’ve learnt how to do that now’
	3.3 Practice integrates the slow thinking style	S8: ‘Sometimes you have to do it 3 or 4 times and try. It takes a while to get off what you are thinking cause the feelings are quite strong’ L18: ‘Instead of just believing and trusting in that fast‐thinking conclusion that I have arrived at, there's been more of an interaction on my part to counteract it with slow thinking… It was much closer to the end of therapy, I was quite actively engaging in slow thinking, quite often’.
4. Value and learning from social connections	4.1 Vignettes and videos help to feel less isolated	L20: ‘I was hearing other people's like feedback and … some of what they said sort of related to me a little bit…it was just helpful because some of the stuff they were saying sort of, it happened to me before … so, yeah it was, I just kind of relate to it sort of’ S1: ‘Well just watching the videos and erm seeing like the people, I wasn't alone, because young people like myself or younger than me or older than me does err have mental health, like I’m not the only one’
	4.2 Learning and support through vignettes and peers	L17:’I’m trying to do what the lady did, he showed me a video of this lady, she has the same problem as me and now when I was watching it, she goes out and comes in and don't let her neighbours worry her and I’m trying to do the same, I’m trying’ S7: ‘[What made the most difference?]’Erm I think the voices of real people. As soon as I left therapy every time, it's stuck in my head you know. So if I get into that situation I try to rethink it the way that SlowMo taught me really, how to do it’ O14: ‘Yeah, so … with the three people, it's a bit limited with what happened to them. I think there is an awful lot more situations that people undergoing problems are confronted with and maybe you could actually put those into the software … expanding it, make it sort of like a broader selection of situations and scenarios’
5. Approaches and challenges of technology	5.1 Use of technology to support positive engagement with the therapy	L15: ‘Before I go out, check phone, then leave, pop the bubbles, slow down. I used it every day, not using it now. The tips helpful and personal message when have worries and messages come up, worked as reminder’ S10: ‘The phone actually helps when you're on the bus … if I start getting agitated about who's looking at me, and who's not looking at me yeah, I just start playing with the bubble’ L16: ‘Every day if I go out, I always do what I need to do on it, like take my deep breaths and get me encouraged to go out… that phone is always with me when I’m out. If I stop, I use it as well’
	5.2 Cognitive demands of blended therapy as therapeutic or overwhelming	O13: ‘You have got someone who is caring and understanding, but then you have got the visual and the video. And the things that might be passing through your mind … you need to sort of hit as many as possible to try and calm me down, so if you have got all of those stimulated by like looking and feeling, and then you are almost touching as well, it's very engaging’ S12: ‘just looking at the computer and erm, and also listening to [therapist], I mean, erm, it was quite, it was understanding what was on the computer with like all the cases they had, of the different [scenarios]… probably nearer to the end, my brain just totally, you know, shut down’
	5.3 Personal relationship with the app	S8: It's difficult for me … yeah I always felt a bit of conscious somebody might be coming along and looking over your shoulder L21: That's right, that is my best friend’ S5: I don't find I need to use it so much now S3: Even though I don't look at the phone, I just remember about slowing down S4: It's a shame that the therapy can't carry on for longer. Once I stopped I felt there was something a bit missing, which is why I’m looking into taking up something now.
	5.4 Challenges to using technology	S9: ‘I have bought things like iPod's and Nintendo but I know the main thing is the smart phone but it's too much technology for me. I can't really be bothered to take the phone out with me’ L18: ‘I had my own phone and I didn't want to go around carrying two phones’ S12: ‘yesterday I tried to look up a few apps on SlowMo and sometimes I was trying to find something about err feeling insecure about something and I tried to find the answer that I gave myself, but I found that a little bit difficult’ L19 –‘umm I did find when I wrote things for the bubbles, the type was a bit big and when the bubbles got smaller I couldn't read everything it said’
6. Improvements in paranoia and well‐being	6.1 Decreases in paranoid thinking and in worry	S10: ‘Well the paranoia has dropped quite a bit yeah. I’m not as extremely paranoid as I used to be’ L21: I’m a worrier, but now I’m not a worrier as much as I was.. it slowed me down quite a lot O13: ‘you do think everybody is talking about you, I mean that was the big thing that I took from it. It just made me think, ‘Hang on a minute, get a grip, it's not like that at all.’ That was the biggest thing that I took from it, just seeing things a bit more clearly’.
	6.2 Increased engagement with social life	L21: ‘I never used to go out, you see, and I go out on my scooter now, with confidence. Before I wouldn't, I always thought people were going to attack me and I don't feel like that now’ L15: I was not going out, not taking bus, at home all the time, walking. After 2/3 months, took the bus. Bike made me feel better and I start working’ O13: ‘I started up my art classes. I used to do a lot on my own, but now I go to other people for more structured art classes’ L18: ‘I suppose my social life has improved. It's made me more comfortable around people. I’ve met more people and done more things I would say. Like just going to the movies, chilling or playing video games’
	6.3 Increased confidence and perseverance	L22: I’m more confident. If I hear something I just brush it under the water, slow thinking for me you know. L17: When I do my housework and they bellow at me, ‘Stop doing, stop doing, we didn't tell you to do it,’ I just carry on doing it. I thought, ‘No, I’m going to carry on doing it.’
	6.4 Support with other mental health difficulties	S12: It's teaching me to not sort of stress so much. Not, you know, not to get over‐anxious about stuff S4: This is the first time in 5 years as I haven't been in hospital with my depression and psychosis. So I think it's really made a difference. Normally I am in hospital 3 or 4 months a year
	6.5 Positive view of the future	S10: ‘Before SlowMo I was doomed yeah but now I’ve got a bit more positive outlook yeah. I can actually live a bit of life’ O13: I was like, ‘I’m a weirdo, a nutter,’ all that kind of thing, and then you realize that there is a lot of people in the same boat, and there is a lot of nice people out there. It just gives you a more positive outlook’

#### STARTING THE SlowMo JOURNEY

People described that the SlowMo therapy was something offered and encouraged by clinicians. It felt like an opportunity to try something new and potentially helpful. It created a mix of emotions. Some people described hope and readiness, especially if they felt they had been stuck with their experiences for a long time. Other people felt nerves and uncertainty about meeting someone new, and if they struggled with voices and anxiety. Importantly, no nerves or uncertainty were expressed in relation to use of technology. For many, this was their first therapy experience.

##### Reasons for Starting SlowMo

Starting SlowMo felt like an opportunity offered by clinicians, as opposed to being sought by patients; as well as being an opportunity for help; and linked to feeling stuck and a desire to take part in research.

##### Feelings before starting

Service users described feeling nervous and uncertain, but also open, willing and ready for new experiences as part of the therapy.

##### Voices and anxiety as barriers

Some symptoms, and specifically voices and anxiety, were barriers to starting SlowMo.

#### CENTRAL ROLE OF SUPPORTIVE THERAPIST RELATIONSHIP

People emphasised the importance of human interaction, of talking and being listened to by someone who was friendly and trusted, as opposed to only interacting with a computer which was thought would be ineffective. The therapist was perceived as crucial in enabling access to both the therapeutic processes and the technology.

##### Importance of talking and being listened to

Some people emphasized the specific value of talking and being heard.

##### A positive therapist relationship

Some people emphasized the importance of non‐specific therapy skills such as warmth, empathy, and trust as part of their positive relationship with the therapist.

##### Therapist as a supportive guide

Some people valued the therapist being flexible, and providing clear explanations, and support to aid engagement with both the therapy and the technology. The therapist supported people to use the technology in and out of the therapy sessions, and the computer content in turn supported the therapist.

#### SLOWING THINGS DOWN

The central concept of slow and fast thinking was helpful; and learning a new skill was valued and integrated into thinking styles, through practice over time; but more cognitively able participants found the intervention to be too slow and proposed that delivery speed be adapted.

##### Slow and Fast thinking are relevant and helpful

Some people related to the idea that they were thinking too quickly, and needed to slow down and not jump to conclusions. Slowing down helped some people to calm down and was useful outside of therapy.

##### Learning a new thinking skill set

Some people described that they had learnt how to slow down their thinking.

##### Practice integrates the slow‐thinking style

Some people described a need to practice the slowthinking style, and to build the habit of slowing their thinking down, over time.

#### VALUE AND LEARNING FROM SOCIAL CONNECTIONS

Some people talked about feeling a connection with the vignette video characters, who were viewed as peers. These connections were normalizing and helped people to feel less isolated. However, the impact went beyond normalizing, in that people described trying to emulate how they had seen the video characters responding to their own experiences.

##### Vignettes and videos help to feel less isolated

People described how they had related to the SlowMo vignettes and learnt that they were not alone, which made them feel less isolated.

##### Learning and support through vignettes and peers

People learnt from and were inspired by the SlowMo vignettes and video stories from peers, and talked about ‘learning from these 3 people.’ They valued the support from others with lived experiences but also wanted a greater range of vignettes.

#### APPROACHES AND CHALLENGES OF TECHNOLOGY

##### Use of technology to support positive engagement with the therapy

The phone app was seen as a tool to aid connectedness in daily‐life; the thought bubble contents were seen as guides to thinking; and the computer scenarios and games were seen as learning tools.

##### Cognitive demands of blended therapy as therapeutic, or overwhelming

For some people the combined cognitive and sensory demands of the blended therapy approach were seen as stimulating and therapeutic, whilst for others these were seen, at times, as overwhelming.

##### Personal relationship with the app

Relationships with the phone app changed over time. Paranoia and self‐consciousness were barriers to use in public for some; the app was described as like a best friend for others, but people gradually moved from the app to memory. For some, the app was insufficient support on its own when therapy ended, and they wanted more face‐to‐face sessions or to take part again.

##### Challenges to using technology

A variety of challenges and issues with technology were described including a lack of interest in technology, limitations of the app interface or due to needing a second phone. Technical issues were noted; and improvements suggested including larger fonts, colour animations, a ‘check in’ rating for how you’re feeling and written instructions on how to use the phone.

#### Improvements in Paranoia and Wellbeing

Different people reported various different ways in which they experienced improvements in their paranoia and wellbeing, which they attributed to the therapy.

##### Decreases in paranoid thinking and in worry

Some people described decreases in paranoia or worry, or described seeing things more clearly.

##### Increased engagement with social life

Some people described that they were able to go out more, take up new leisure activities, see more people and generally increase their engagement with their social life following the therapy.

##### Increased confidence and perseverance

Others described feeling more confident and determined to get on with their lives.

##### Support with other mental health difficulties

People also described impacts on a variety of other mental health issues including anxiety, stress, panic attacks, voice hearing, and depression.

##### Positive view of the future

Finally, several people talked about having a more positive outlook as a result of the therapy.

## DISCUSSION

This study provided an in‐depth exploration of the subjective user experience of SlowMo therapy and generated insights to aid future development and support implementation. The 6 core themes captured (i) the experiences of starting the SlowMo journey; (ii) the central role of the supportive therapist as a guide to accessing the therapy and technology; (iii) the approaches and challenges in the use of the technology; (iv) key features of the therapy including slowing things down, which was learnt and internalized through time and practice; and (v) the value and learning achieved through relating to the vignette characters, which helped to normalize experiences and model coping strategies. Finally, (vi) positive impacts of the SlowMo therapy were reported for paranoia, worry, other mental health difficulties, engagement with social and daily life, confidence, and a positive outlook for the future.

Primary reasons for starting the SlowMo therapy were often a sense of feeling stuck and an opportunity for help. One peer researcher noted that ‘people on the SlowMo trial had not generally been offered any other therapies’. Some of the quotes make it clear that problems had never been discussed before.

Consistent with other blended therapies (Vernmark et al., 2019), the presence of the therapist during the session was viewed as central to the process. The therapist was seen as crucial in bridging the link between the service user and the technical features of the app, which in turn led to more favourable experiences of the technology, cementing the triangle of alliance (Cavanagh, 2010). The majority opinion was that the digital component of the therapy augmented the rich therapeutic relationship, as opposed to the app being seen as central (Aref‐Adib et al., 2019). This is supported by a recent systematic review which found that when peer‐to‐peer interactions on mHealth apps were not moderated by clinicians or researchers, the retention rates slipped from very high (94.5%) to very low (14%), highlighting the role of the therapist in improving adherence (Biagianti et al., 2017). Importantly in the current study, participants also described a relationship with the phone app itself. The app reminded participants of the learning gained in therapy and became a ‘best friend’ for one person.

As therapy progressed, multiple participants in this sub‐study, found that the interactive multimedia features of the vignettes made them feel more connected and understood, and less isolated in their experiences. Studies have shown that participants enjoy digital platforms which are tailored, personalized, and communicative (Berry et al., 2016). Some participants learned and retained their therapy skills better by observing the animated vignettes, and several participants requested further tailoring through a greater variety of characters, covering a broader set of worries that occur in paranoia. This experience of viewing others who are coping with similar experiences to oneself is an important factor that enhances acceptability and engagement with interventions (Biagianti et al., 2018).

Many participants recognized the importance of slowing down thinking and were able to modify their thinking style to be more flexible and to seek more information. This is consistent with the main RCT finding that the SlowMo therapy increased belief flexibility and slow thinking compared to treatment as usual (Garety et al., 2021).

The within‐app thought bubbles helped to develop skills to slow down thinking, and were an accessible resource that offered coping strategies. Participants were more likely to use the app earlier in the therapy, when paranoia was arguably more acute, with use appearing to reduce as participants’ mental state improved, and new thinking processes became internalized. This is consistent with the concept of e‐attainment of personal goals when these have been sufficiently supported (Sanatkar et al., 2019).

While SlowMo offers a normalizing, supportive platform for participants, some described that suspiciousness and pre‐existing views towards technology might affect use. This is consistent with previous reports (Aref‐Adib et al., 2019), although importantly, baseline paranoia did not predict adherence to or self‐reported user experiences of the SlowMo app (Hardy et al., 2022). The stigma surrounding mental health continued to be a barrier, especially for people suffering from paranoia, who on occasion felt self‐conscious when using the app in public. Participants also described technical challenges, as expected given the trial tested a working prototype (minimal viable product) of SlowMo therapy (Hardy et al., 2018). Technical recommendations have been integrated into a product specification for an updated version of SlowMo therapy; including improved syncing of content from computer to the mobile app, increased font size, additional instruction guide (on‐boarding) for use of the mobile phone app, and coding to enable cross‐platform use of the mobile app. These recommendations are consistent with previously published recommendations to support the implementation of digital health interventions, whereby interventions that are adaptable to users’ needs and available on participants’ own phones led to better engagement (Aref‐Adib et al., 2019).

Lastly, participants reported improvements in paranoia, worry, confidence, distress, outlook, and social life, mirroring the quantitative results from the main trial which showed positive impacts of the therapy on paranoia and worry alongside other well‐being, self‐concept, and quality of life outcomes (Garety et al., 2021). The therapy proved to have far‐reaching impacts: participants mentioned enhanced social lives, and improved co‐existing conditions, in keeping with the broad pattern of improvements across secondary outcomes reported in the main trial. Participants reported increased self‐confidence and better management of other stresses, which is consistent with the use of technology to promote a sense of autonomy, and for mobile phone apps to offer real‐time help (Bucci et al., 2018a).

To conclude, participants of the SlowMo therapy reported a positive experience of the digitally supported therapy, reductions in paranoia, and improved overall well‐being. This was linked to feeling understood, relating to vignettes, accepting and learning a new slow thinking style, and being supported by a therapist to reinforce their learning through the therapy sessions and mobile phone app use.

### Strengths and limitations

This is the first study to gather detailed personal and contextual information to aid understanding of the factors influencing subjective user‐experience of a specific blended digital therapy for paranoia. The data were gathered from a representative sample of over 12% of all participants randomized to SlowMo therapy. Peer researchers co‐delivered the study, conducted all interviews with participants, and co‐produced the final thematic analysis, which it is hoped, enhanced the robustness of the results and the validity of the service user perspective. A number of steps were taken during the course of the analysis to enhance the overall rigor of the study and limit sources of bias. The initial coding was conducted by an independent researcher, with multiple coding and triangulation to reach a consensus on the theme structure. Regular meetings involved a diverse mix of professionals, and peer researchers to ensure that participants’ responses were considered from a variety of viewpoints.

The participants recruited in previous qualitative studies (e.g. Aref‐Adib et al., 2016; Bucci et al., 2018b) were mostly young, digital‐natives with a mean age of 28 and 26 years, respectively. A systematic review found that younger ‘at risk’ or first‐episode psychosis individuals used the internet and mobile technologies for their mental health difficulties more frequently than those with longer‐term psychosis (Killikelly et al., 2017). The mean age of SlowMo participants was 45, thus a notable aspect of the current study was the broadly positive experience of blended therapy in this older age group. This is further supported by a linked study that showed no impact of the digital divide, as neither age, ethnicity nor paranoia impacted adherence to or user experience of the SlowMo mobile app (Hardy et al., 2022).

Limitations included the limited sampling of participants from one site, and limited ethnic diversity, in this qualitative study, especially from black ethnic groups, who reported lower computer access, smartphone use, and confidence prior to therapy in a linked study (Hardy et al., 2022). In addition, only 4 participants (18%) were female. The diversity of the sample with respect to digital literacy, digital access, socioeconomic status, and cognitive ability was not investigated in this qualitative study, although it was investigated in the linked SlowMo study (Hardy et al., 2022). Although participants were invited consecutively, it is unclear whether those who agreed to take part were those who were more positive about the therapy. Finally, the derived themes reflected a majority view of the participants in this study but did not necessarily reflect the views of all participants in this study or indeed in the main trial.

## CONCLUSIONS

These findings offer valuable insights into participants’ experiences with SlowMo therapy. Importantly, many participants described feeling stuck prior to starting the therapy. They valued the central role of the supportive therapist who guided them through the therapy, and the use of the technology. They described that key concepts of slow and fast thinking were helpful, and they valued learning from the connections with the vignette characters. The relationship with the mobile phone app was experienced positively. Furthermore, the integration of the slow thinking style over time in a form of e‐attainment (whereby technology has sufficiently supported the attainment of therapy goals or skills) may have contributed to the subjective improvements in paranoia, well‐being, and social integration, and reductions in worry, stress, and depression. In this respect, the ‘triangle of alliance’ between service users, therapists, and digital platforms appeared to be highly effective, and there was a clear sense of a shared bond, goals, and tasks to support improved paranoia and mental well‐being. In the future, blended therapies for psychosis may benefit from a wide variety of true stories and experiences on which service users’ could model their recovery, to optimize impact. Important reflections were provided on the less visible personal and social barriers that affect the uptake of digital therapies such as the need for further instructions on how to use mobile phone apps, preferences for using apps on one's own phone, and the impact on usage pattern when symptoms become acute. This study in combination with the quantitative user experience study (Hardy et al., 2022) provides critical information to support the development of the next iteration of the SlowMo and other blended therapies for future implementation in clinical practice.

## AUTHOR CONTRIBUTIONS

Garety, Ward, Greenwood, Hardy, Emsley, Freeman, Fowler, Kuipers, Bebbington and Dunn were responsible for concept and design of the study and securing funding. Greenwood, Gurnani, Ward, Hardy, Vogel, Vella, McGourty, Robertson, Sacadura, Rus‐Calafell, Collett, Michelson and the Patient and Public Involvement team were responsible for acquisition, analysis and interpretation of the data. Greenwood, Gurnani, Garety, Ward, Hardy and Michelson were responsible for the drafting of the manuscript, and all authors provided critical revision of the manuscript for important intellectual content.

## Supporting information

Supplementary Material

## Data Availability

The data that support the findings of this study are available from the corresponding author upon reasonable request. The data are not publicly available due to privacy or ethical restrictions.

## APPENDIX 1

### The slowmo experience: semi‐structured interview topic guide

GUIDANCE FOR PPI RESEARCHERS. Below is an introduction to the interview and a set of questions for you to ask. All of the questions are guidance for what you might say to a service user in an interview. The bold questions are important to ask and the remaining questions are prompts that you might find helpful, dependent on what the person has already told you.

INTRODUCTIONS: The following questions ask for your opinions about what it was like for you to attend SlowMo therapy sessions with [therapist name]. Your responses will be used to help us to understand more about what it is like to do this new therapy and how it might be improved. So first we're just going to talk in general about SlowMo therapy.

### Warm‐up Question

Could you tell me a little about your reasons for/why you decided to take part in the SlowMo therapy?

- *Why did you take it up now?*

How were you feeling when you thought of starting your first sessions? [Explore: positive feelings/ worries/anxiety?]

### Use of technology in session (computer) / outside sessions (mobile phone app)

How did you find using the computer in sessions?

- In what ways was it helpful/less helpful?

So the therapy gave you a smart phone, how did you feel about using it? Did you use it?

- If yes why (positive feelings) and if no, why not? What put you off or stopped you (worries/concerns/suspicions)?
- How did you feel about using a smartphone (if you don't have one) or the SlowMo phone (if you do have one)?

Did you use the SlowMo app on the phone?

- If yes: how did you find it? How often did you use it? Are you still using it?
- In what ways was it less helpful?
- Were there any barriers/obstacles to using the app? (practical worries/technology worries)

How could your experience of using the computer / mobile phone app be improved?

### Your Therapist

How did you find working with the therapist?

- How important was the relationship with the therapist?

How could your therapist improve the experience of therapy?

### Reflections on Therapy Sessions

What did you think about the SlowMo approach overall?

What did you think about the focus on fast and slow thinking?

- How relevant was it to your worries?
- What did you think about the interactive stories and games? the amount of sessions and how long they lasted for?

Have you noticed any changes in dealing with your worries since having the therapy?

Is there anything that you're still using from the therapy, in your daily life? If so, what?

### Final Questions / Experience of Taking Part

Would you make any changes to…

- The computer app? The mobile phone app?
- Is there anything missing? Is there anything you would change?

Looking back at the therapy, what made the most difference for you?

Was there anything you particularly enjoyed or didn't like about SlowMo therapy?

How do you see things now that you've had therapy?

What would you say to someone who thinking about trying SlowMo therapy?

## References

[papt12393-bib-0001] Allan, S. , Bradstreet, S. , Mcleod, H. , Farhall, J. , Lambrou, M. , Gleeson, J. , Clark, A. , & Gumley, A. (2019). EMPOWER group, gumley A developing a hypothetical implementation framework of expectations for monitoring early signs of psychosis relapse using a mobile app: Qualitative study. J Med Internet Res, 21(10), e14366. 10.2196/14366 31651400 PMC6838692

[papt12393-bib-0002] Aref‐Adib, G. , McCloud, T. , Ross, J. , O’Hanlon, P. , Appleton, V. , Rowe, S. , & Lobban, F. (2019). Factors affecting implementation of digital health interventions for people with psychosis or bipolar disorder, and their family and friends: A systematic review. The Lancet Psychiatry, 6(3); 257–266. 10.1016/S2215-0366(18)30302-X 30522979

[papt12393-bib-0003] Aref‐Adib, G. , O’Hanlon, P. , Fullarton, K. , Morant, N. , Sommerlad, A. , Johnson, S. , & Osborn, D. (2016). A qualitative study of online mental health information seeking behaviour by those with psychosis. BMC Psychiatry, 16(1), 232. 10.1186/s12888-016-0952-0 27400874 PMC4940927

[papt12393-bib-0004] Ben‐Zeev, D. , Brenner, C.J. , Begale, M. , Duffecy, J. , Mohr, D.C. , & Mueser, K.T. (2014). Feasibility, acceptability, and preliminary efficacy of a smartphone intervention for schizophrenia. Schizophrenia Bulletin, 40(6), 1244–1253. 10.1093/schbul/sbu033 24609454 PMC4193714

[papt12393-bib-0005] Berry, N. , Lobban, F. , & Bucci, S. (2019). A qualitative exploration of service user views about using digital health interventions for self‐management in severe mental health problems. BMC Psychiatry, 19, 35. 10.1186/s12888-018-1979-1 30665384 PMC6341527

[papt12393-bib-0006] Berry, N. , Lobban, F. , Emsley, R. , & Bucci, S. (2016). Acceptability of interventions delivered online and through mobile phones for people who experience severe mental health problems: A systematic review. Journal of Medical Internet Research, 18(5), e121. 10.2196/jmir.5250 27245693 PMC4908305

[papt12393-bib-0007] Bhaskar, R. (2009). Scientific realism and human emancipation. Routledge.

[papt12393-bib-0008] Biagianti, B. , Hidalgo‐Mazzei, D. , & Meyer, N. (2017). Developing digital interventions for people living with serious mental illness: Perspectives from three mHealth studies. Evidence Based Mental Health, 20(4), 98–101. 10.1136/eb-2017-102765 29025862 PMC5750413

[papt12393-bib-0009] Biagianti, B. , Quraishi, S.H. , & Schlosser, D.A. (2018). Potential benefits of incorporating peer‐to‐peer interactions into digital interventions for psychotic disorders: A systematic review. Psychiatric Services, 69(4), 377–388. 10.1176/appi.ps.201700283 29241435 PMC5988432

[papt12393-bib-0010] Bighelli, I. , Salanti, G. , Huhn, M. , Schneider‐Thoma, J. , Krause, M. , Reitmeir, C. , Wallis, S. , Schwermann, F. , Pitschel‐Walz, G. , Barbui, C. , Furukawa, T.A. , & Leucht, S. (2018). Psychological interventions to reduce positive symptoms in schizophrenia: systematic review and network meta‐analysis. World Psychiatry, 17(3), 316–329. 10.1002/wps.20577 30192101 PMC6127754

[papt12393-bib-0011] Bordin, E.S. (1979). The generalizability of the psychoanalytic concept of the working alliance. Psychotherapy: Theory, Research & Practice, 16(3), 252–260.

[papt12393-bib-0012] Braun, V. , & Clarke, V. (2006). Using thematic analysis in psychology. Qualitative Research in Psychology, 3(2), 77–101. 10.1191/1478088706qp063oa

[papt12393-bib-0013] Bucci, S. , Barrowclough, C. , Ainsworth, J. , Machin, M. , Morris, R. , Berry, K. , & Haddock, G. (2018a). Actissist: Proof‐of‐concept trial of a theory‐driven digital intervention for psychosis. Schizophrenia Bulletin, 44(5), 1070–1080. 10.1093/schbul/sby0 29566206 PMC6135229

[papt12393-bib-0014] Bucci, S. , Morris, R. , Berry, K. , Berry, N. , Haddock, G. , Barrowclough, C. , Lewis, S. , & Edge, D. (2018b). Early psychosis service user views on digital technology: Qualitative analysis. JMIR Mental Health, 5(4), e10091. 10.2196/10091 30381280 PMC6236205

[papt12393-bib-0015] Cavanagh, K. (2010). Turn on, tune in, don’t drop out: uptake, engagement and disengagement with internet based CBT. In J. Bennett‐Levy et al (Eds.) Oxford Guide to Low‐Intensity CBT Interventions, pp. 227–234. Oxford University Press.

[papt12393-bib-0016] Cavanagh, K. , Herbeck Belnap, B. , Rothenberger, S.D. , Abebe, K.Z. , & Rollman, B.L. (2018). My care manager, my computer therapy and me: The relationship triangle in computerized cognitive behavioural therapy. Internet Interventions, 11, 11–19. 10.1016/j.invent.2017.10.005 30135755 PMC6084903

[papt12393-bib-0017] Clarke, J. , Proudfoot, J. , Whitton, A. , Birch, M.‐R. , Boyd, M. , Parker, G. , Manicavasagar, V. , Hadzi‐Pavlovic, D. , & Fogarty, A. (2016). Therapeutic alliance with a fully automated mobile phone and web‐based intervention: Secondary analysis of a randomized controlled trial. JMIR Mental Health, 3, e10. 10.2196/mental.4656 26917096 PMC4786687

[papt12393-bib-0018] Coid, J.W. , Ullrich, S. , Kallis, C. , Keers, R. , Barker, D. , Cowden, F. , & Stamps, R. (2013). The relationship between delusions and violence: Findings from the east London first episode psychosis Study. JAMA Psychiatry, 70(5), 465–471. 10.1001/jamapsychiatry.2013.12 23467760

[papt12393-bib-0019] Collier, A. (1994). Critical realism: an introduction to Roy Bhaskar's philosophy. Verso.

[papt12393-bib-0020] D'Alfonso, S. , Lederman, R. , Bucci, S. , & Berry, K. (2020). The digital therapeutic alliance and human‐computer interaction. JMIR Ment Health, 7(12), e21895. 10.2196/21895 33372897 PMC7803473

[papt12393-bib-0021] Freeman, D. , Dunn, G. , Startup, H. , Pugh, K. , Cordwell, J. , Mander, H. , Černis, E. , Wingham, G. , Shirvell, K. , & Kingdon, D. (2015a). Effects of cognitive behaviour therapy for worry on persecutory delusions in patients with psychosis (WIT): A parallel, single‐blind, randomised controlled trial with a mediation analysis. The Lancet Psychiatry, 2(4), 305–313. 10.1016/S2215-0366(15)00039-5 26360083 PMC4698664

[papt12393-bib-0022] Freeman, D. , & Garety, P. (2006). Helping patients with paranoid and suspicious thoughts: A cognitive–behavioural approach. Advances in Psychiatric Treatment, 12(6), 404–415. 10.1192/apt.12.6.404

[papt12393-bib-0023] Freeman, D. , McManus, S. , Brugha, T. , Meltzer, H. , Jenkins, R. , & Bebbington, P. (2011). Concomitants of paranoia in the general population. Psychological Medicine, 41(5), 923–936. 10.1017/S0033291710001546 20735884

[papt12393-bib-0024] Freeman, D. , Pugh, K. , Dunn, G. , Evans, N. , Sheaves, B. , Waite, F. , Černis, E. , Lister, R. , & Fowler, D. (2014). An early Phase II randomised controlled trial testing the effect on persecutory delusions of using CBT to reduce negative cognitions about the self: The potential benefits of enhancing self confidence. Schizophrenia Research, 160(1–3), 186–192. 10.1016/j.schres.2014.10.038 25468186 PMC4266450

[papt12393-bib-0025] Freeman, D. , Waite, F. , Startup, H. , Myers, E. , Lister, R. , McInerney, J. , Harvey, A.G. , Geddes, J. , Zaiwalla, Z. , Luengo‐Fernandez, R. , Foster, R. , Clifton, L. , & Yu, L.‐M. (2015b). Efficacy of cognitive behavioural therapy for sleep improvement in patients with persistent delusions and hallucinations (BEST): A prospective, assessor‐blind, randomised controlled pilot trial. Lancet Psychiatry, 2, 975–983. 10.1016/S2215-0366(15)00314-4 26363701 PMC4641164

[papt12393-bib-0026] Garety, P.A. , & Freeman, D. (2013). The past and future of delusions research: From the inexplicable to the treatable. British Journal of Psychiatry, 203(5), 327–333. 10.1192/bjp.bp.113.126953 24187067

[papt12393-bib-0027] Garety, P.A. , Freeman, D. , Jolley, S. , Dunn, G. , Bebbington, P.E. , Fowler, D.G. , Kuipers, E. , & Dudley, R. (2005). Reasoning, emotions, and delusional conviction in psychosis. Journal of Abnormal Psychology, 114(3), 373–384. 10.1037/0021-843X.114.3.373 16117574

[papt12393-bib-0028] Garety, P. , Waller, H. , Emsley, R. , Jolley, S. , Kuipers, E. , Bebbington, P. , Dunn, G. , Fowler, D. , Hardy, A. , & Freeman, D. (2015). Cognitive mechanisms of change in delusions: An experimental investigation targeting reasoning to effect change in paranoia. Schizophrenia Bulletin, 41(2), 400–410. 10.1093/schbul/sbu103 25053650 PMC4332945

[papt12393-bib-0029] Garety, P.A. , Ward, T. , Emsley, R. , Greenwood, K. , Freeman, D. , Fowler, D. , Kuipers, E. , Bebbington, P. , Rus‐Calafell, M. , McGourty, A. , Sacadura, C. , Collett, N. , James, K. , & Hardy, A. (2021). Effects of SlowMo, a blended digital therapy targeting reasoning, on paranoia among people with psychosis: A randomized clinical trial. JAMA Psychiatry, 78(7), 714. 10.1001/jamapsychiatry.2021.0326 33825827 PMC8027943

[papt12393-bib-0030] Garety, P.A. , Ward, T. , Freeman, D. , Fowler, D. , Emsley, R. , Dunn, G. , & Hardy, A. (2017). SlowMo, a digital therapy targeting reasoning in paranoia, versus treatment as usual in the treatment of people who fear harm from others: Study protocol for a randomised controlled trial. Trials, 18(1), 510. 10.1186/s13063-017-2242-7 29096681 PMC5667466

[papt12393-bib-0031] Gillard, S. , Borschmann, R. , Turner, K. , Goodrich‐Purnell, N. , Lovell, K. , & Chambers, M. (2010). ‘What difference does it make?’ Finding evidence of the impact of mental health service user researchers on research into the experiences of detained psychiatric patients. Health Expectations, 13(2), 185–194. 10.1111/j.1369-7625.2010.00596.x 20536538 PMC5060533

[papt12393-bib-0032] Gillard, S. , Simons, L. , Turner, K. , Lucock, M. , & Edwards, C. (2012). Patient and public involvement in the coproduction of knowledge: Reflection on the analysis of qualitative data in a mental health study. Qualitative Health Research, 22(8), 1126–1137. 10.1177/1049732312448541 22673090

[papt12393-bib-0033] Goldsmith, L.P. , Lewis, S.W. , Dunn, G. , & Bentall, R.P. (2015). Psychological treatments for early psychosis can be beneficial or harmful, depending on the therapeutic alliance: An instrumental variable analysis. Psychological Medicine, 2015(45), 2365–2373. 10.1017/S003329171500032X PMC4501302 25805118

[papt12393-bib-0034] Green, C. E. , Freeman, D. , Kuipers, E. , Bebbington, P. , Fowler, D. , Dunn, G. , & Garety, P. A. (2008). Measuring ideas of persecution and social reference: The Green et al, Measuring ideas of persecution and social reference: The Green. Psychological Medicine, 38, 101–111. 10.1017/S0033291707001638 17903336

[papt12393-bib-0035] Greenwood, K. , Alford, K. , O'Leary, I. , Peters, E. , Hardy, A. , Cavanagh, K. , Field, A.P. , de Visser, R. , Fowler, D. , Davies, M. , Papamichail, A. , & Garety, P. (2018). The U&I study: Study protocol for a feasibility randomised controlled trial of a pre‐cognitive behavioural therapy digital ‘informed choice’ intervention to improve attitudes towards uptake and implementation of CBT for psychosis. Trials, 19(1), 644. 10.1186/s13063-018-3023-7 30458850 PMC6247503

[papt12393-bib-0036] Greenwood, K. , Robertson, S. , Vogel, E. , Vella, C. , Ward, T. , McGourty, A. , Sacadura, C. , Hardy, A. , Rus‐Calafell, M. , Collett, N. , Emsley, R. , Freeman, D. , Fowler, D. , Kuipers, E. , Bebbington, P. , Dunn, G. , Garety, P. & and the SLoMo Patient and Public Involvement (PPI) team (2021). The impact of patient and public involvement in the slowmo study: Reflections on peer innovation. Health expectations. Health Expectations, 25(1), 191–202. 10.1111/hex.13362 34585482 PMC8849241

[papt12393-bib-0037] Hammersley, M. (2004). Action research: A contradiction in terms? Oxford Review of Education, 30(2), 165–181. 10.1080/0305498042000215502

[papt12393-bib-0038] Hardy, A. , Ward, T. , Emsley, R. , Greenwood, K. , Freeman, D. , Fowler, D. , Kuipers, E. , Bebbington, P. , & Garety, P. (2022). Bridging the ‘digital divide’ in psychological therapies for paranoia in psychosis: The user experience of the SlowMo mobile app. Under review. 10.2196/29725 PMC9288106 35776506

[papt12393-bib-0039] Hardy, A. , Wojdecka, A. , West, J. , Matthews, E. , Golby, C. , Ward, T. , Lopez, N.D. , Freeman, D. , Waller, H. , Kuipers, E. , Bebbington, P. , Fowler, D. , Emsley, R. , Dunn, G. , & Garety, P. (2018). How inclusive, user‐centered design research can improve psychological therapies for psychosis: Development of SlowMo. JMIR Ment Health, 5(4), e11222. 10.2196/11222 30518514 PMC6300708

[papt12393-bib-0040] Henson, P. , Wisniewski, H. , Hollis, C. , Keshavan, M. , & Torous, J. (2019). Digital mental health apps and the therapeutic alliance: initial review. BJPsych Open, 5(1), e15. 10.1192/bjo.2018.86 30762511 PMC6381418

[papt12393-bib-0041] Holloway, I. , & Todres, L. (2003). The status of method: Flexibility, consistency and coherence. Qualitative Research, 3(3), 345–357. 10.1177/1468794103033004

[papt12393-bib-0042] Johns, L.C. , Cannon, M. , Singleton, N. , Murray, R.M. , Farrell, M. , Brugha, T. , Bebbington, P. , Jenkins, R. , & Meltzer, H. (2004). Prevalence and correlates of self‐reported psychotic symptoms in the British population. British Journal of Psychiatry, 185, 298–305. 10.1192/bjp.185.4.298 15458989

[papt12393-bib-0043] Killikelly, C. , He, Z. , Reeder, C. , & Wykes, T. (2017). Improving adherence to web‐based and mobile technologies for people with psychosis: Systematic review of new potential predictors of adherence. JMIR MHealth and UHealth, 5(7), e94. 10.2196/mhealth.7088 28729235 PMC5544896

[papt12393-bib-0044] Kooistra, L.C. , Ruwaard, J.E. , Wiersma, P. , van Oppen, R. , van der Vaart, J.E.W.C. , & van Gemert‐Pijnen, R.H. (2016). Development and initial evaluation of blended cognitive behavioural treatment for major depression in routine specialized mental health care. Internet Interventions, 4, 61–71. 30135791 10.1016/j.invent.2016.01.003 PMC6096194

[papt12393-bib-0045] Letourneau, N. , & Allen, M. (2006). Post‐positivistic critical multiplism: A beginning dialogue. In W.K. Cody (Ed.), Philosophical and theoretical perspectives for advanced nursing practice (pp. 221–231). Jones and Bartlett Publishers. 10.1046/j.1365-2648.1999.01133.x 10499219

[papt12393-bib-0046] Lopez, A. (2015). An investigation of the use of Internet based resources in support of the therapeutic alliance. Clinical Social Work Journal, 43, 189–200. 10.1007/s10615-014-0509-y

[papt12393-bib-0047] Lopez, A. , Schwenk, S. , Schneck, C.D. , Griffin, R.J. , & Mishkind, M.C. (2019). Technology‐based mental health treatment and the impact on the therapeutic alliance. Current Psychiatry Reports, 21, 76. 10.1007/s11920-019-1055-7 31286280

[papt12393-bib-0048] Ng, M.M. , Firth, J. , Minen, M. , & Torous, J. (2019). User engagement in mental health apps: A review of measurement, reporting, and validity. Psychiatric Services, 2019(70), 538–544. 10.1176/appi.ps.201800519 PMC6839109 30914003

[papt12393-bib-0049] Nowell, L.S. , Norris, J.M. , White, D.E. , & Moules, N.J. (2017). Thematic analysis: Striving to meet the trustworthiness criteria. International Journal of Qualitative Methods, 16(1), 1609406917733847. 10.1177/1609406917733847

[papt12393-bib-0050] Ponterotto, J.G. (2005). Qualitative research training in counseling psychology: A survey of directors of training. Teaching of Psychology, 32(1), 60–62.

[papt12393-bib-0051] Porter, S. (2007). Validity, trustworthiness and rigour: reasserting realism in qualitative research. Journal of Advanced Nursing, 60(1), 79–86. 10.1111/j.1365-2648.2007.04360.x 17824942

[papt12393-bib-0052] Robotham, D. , Satkunanathan, S. , Doughty, L. , & Wykes, T. (2016). Do we still have a digital divide in mental health? A five‐year survey follow‐up. Journal of Medical Internet Research, 18(11), e309. 10.2196/jmir.6511 27876684 PMC5141335

[papt12393-bib-0053] Sanatkar, S. , Baldwin, P.A. , Huckvale, K. , Clarke, J. , Christensen, H. , Harvey, S. , & Proudfoot, J. (2019). Using cluster analysis to explore engagement and e‐attainment as emergent behavior in electronic mental health. Journal of Medical Internet Research, 21(11), e14728. 10.2196/14728 31778115 PMC6908978

[papt12393-bib-0054] Sarkar, U. , Gourley, G.I. , Lyles, C.R. , Tieu, L. , Clarity, C. , Newmark, L. , Singh, K. , & Bates, D.W. (2016). Usability of commercially available mobile applications for diverse patients. Journal of General Internal Medicine, 31, 1417–1426. 10.1007/s11606-016-3771-6 27418347 PMC5130945

[papt12393-bib-0055] Simpson, E. , & House, A.O. (2002). Involving users in the delivery and evaluation of mental health services: Systematic review. British Medical Journal, 325, 1265. 12458241 10.1136/bmj.325.7375.1265 PMC136921

[papt12393-bib-0056] Vernmark, K. , Hesser, H. , Topooco, N. , Berger, T. , Riper, H. , Luuk, L. , Backlund, L. , Carlbring, P. , & Andersson, G. (2019). Working alliance as a predictor of change in depression during blended cognitive behaviour therapy. Cognitive Behaviour Therapy, 48(4), 285–299. 10.1080/16506073.2018.1533577 30372653

[papt12393-bib-0057] Ward, T. , & Garety, P.A. (2017). Fast and slow thinking in distressing delusions: A review of the literature and implications for targeted therapy. Schizophrenia Research, 203, 80–87. 10.1016/j.schres.2017.08.045 28927863 PMC6336980

[papt12393-bib-0058] World Health Organisation (2010). The ICD‐10 classification of mental and behavioural disorders: Diagnostic criteria for research. WHO.

[papt12393-bib-0059] World Health Organization (1992). SCAN Schedules for clinical assessment in neuropsychiatry, version 1.0. World Health Organization.

