# Supplementary material for: The service user experience of SlowMo therapy: A co‐produced thematic analysis of service users’ subjective experience
Source: Psychol Psychother. 2022 Apr 20;95(3):680–700. doi: 10.1111/papt.12393 (PMC9873386; doi:10.1111/papt.12393)
Supplement: Supplementary file 1 — Supplementary Material [file PAPT-95-680-s001.pdf]

Supporting information

THE SLOWMO EXPERIENCE: SEMI-STRUCTURED INTERVIEW TOPIC GUIDE GUIDANCE FOR PPI RESEARCHERS.

Below is an introduction to the interview and a set of questions for you to ask. All of the questions are guidance for what you might say to a service user in an interview.

The bold questions are important to ask and remaining questions are prompts that you might find helpful, dependent on what the person has already told you.

INTRODUCTIONS: The following questions ask for your opinions about what it was like for you to attend SlowMo therapy sessions with [therapist name]. Your responses will be used to help us to understand more about what it is like to do this new therapy and how it might be improved. So first we're just going to talk in general about the SlowMo therapy.

Warm-up Question

Could you tell me a little about your reasons for/why you decided to take part in the SlowMo therapy?

- *Why did you take it up now?*

How were you feeling when you thought of starting your first sessions? [Explore: positive feelings/worries/anxiety?]

Use of technology in session (computer) / outside sessions (mobile phone app)

How did you find using the computer in sessions?

- ***In what ways was it helpful/less helpful?* So the therapy gave you a smart phone, how did you feel about using it? Did you use it?**

- *If yes why (positive feelings) and if no, why not? What put you off or stopped you (worries/concerns/suspicions)?*

- *How did you feel about using a smartphone (if you don't have one) or the SlowMo phone (if you do have one)?*

Did you use the SlowMo app on the phone?
- *If yes: how did you find it? How often did you use it? Are you still using it?*
- *In what ways was it less helpful?*
- *Were there any barriers/obstacles to using the app? (practical worries/technology worries)*

How could your experience of using the computer / mobile phone app be improved?

Your Therapist

How did you find working with the therapist?
- *How important was the relationship with the therapist?*

How could your therapist improve the experience of therapy?
Reflections On Therapy Sessions

What did you think about the SlowMo approach overall?

What did you think about the focus on fast and slow thinking?
- *How relevant was it to your worries?*
- *What did you think about the interactive stories and games? the amount of sessions and how long they lasted for?*

Have you noticed any changes in dealing with your worries since having the therapy? Is there anything that you're still using from the therapy, in your daily life? If so, what?

Final Questions / Experience Of Taking Part
Would you make any changes to…

- **The computer app? The mobile phone app?**
- Is there anything missing? Is there anything you would change?

Looking back at the therapy, what made the most difference for you?

Was there anything you particularly enjoyed or didn't like about SlowMo therapy?

How do you see things now that you've had therapy?

What would you say to someone who thinking about trying SlowMo therapy?
